# The Biological Active Substances of *Taraxacum officinale* and *Arctium lappa* from the Siberian Federal District

**DOI:** 10.3390/ijms25063263

**Published:** 2024-03-13

**Authors:** Anna S. Frolova, Anna D. Fokina, Irina S. Milentyeva, Lyudmila K. Asyakina, Larisa A. Proskuryakova, Alexander Y. Prosekov

**Affiliations:** 1 Laboratory of Biotesting of Natural Nutraceuticals of Scientific and Innovative Management, Kemerovo State University, 6, Krasnaya St., Kemerovo 650000, Russia; frolova.anna.s@mail.ru (A.S.F.); fadan-2001@mail.ru (A.D.F.); irazumnikova@mail.ru (I.S.M.); 2 Laboratory of Phytoremediation of Technogenically Disturbed Ecosystems, Kemerovo State University, 6, Krasnaya St., Kemerovo 650000, Russia; 3 Scientific and Innovative Management Department, Kemerovo State University, 6, Krasnaya St., Kemerovo 650000, Russia; lora-al@yandex.ru; 4 Department of Bionanotechnology, Kemerovo State University, 6, Krasnaya St., Kemerovo 650000, Russia; rector@kemsu.ru

**Keywords:** dandelion root, burdock root, raw material, antioxidants, flavonoids, bioactive substances, vitamins, *Escherichia coli*

## Abstract

Currently, scientists are increasingly focusing on utilizing the natural flora of the planet to search for and isolate individual bioactive substances that prevent various diseases, contribute to increased life expectancy, and affect all major life-supporting systems in the human body. This study describes the examination of the composition of plant raw materials from the Siberian Federal District. The research focuses on plant specimens from the root parts of *Taraxacum officinale* and *Arctium lappa*, collected in the Kemerovo region. The study determines the contents of the water-soluble vitamins B and C in the research subjects. The investigation includes assessing antioxidant properties, antimicrobial activity, and flavonoid content in extracts based on plant raw materials. All samples show a high percentage of antioxidant activity, with the highest antioxidant activity for *T. officinale* at 85.51 and that for *A. lappa* at 88.97. The results indicate low antimicrobial activity against *Escherichia coli* (growth inhibition zone up to 15.5 mm). Plant extracts contain significant amounts of B-group vitamins, with pyridoxine in *T. officinale* (156.40 μg/mL) and thiamine (46.20 μg/mL) and pyridoxine (357.10 μg/mL) in *Arctium lappa*. Flavonoids (rutin and quercetin) are identified in *T. officinale* and *A. lappa* extracts based on the study results.

## 1. Introduction

The resources that our planet is rich in are dwindling, such as valuable minerals, forests, and naturally sourced food products. Climate conditions are becoming increasingly unpredictable each year. Population density and growth are on the rise.

According to United Nations projections, the world’s population will reach 8.5 billion people by 2030, 9.7 billion by 2050, and 10.4 billion by 2100 [1]. Consequently, humanity faces pressing questions about finding new sources of food, achieving self-sufficiency in providing necessary food products, and strengthening population immunity against infections and pathogens using Russian foods, medicines, and functional additives to the basic human diet.

The Siberian Federal District offers a rich diversity of plant materials with varied phytochemical compositions and properties [2]. Currently, the study of individual bioactive substances (BAS) is a relevant field in biotechnology science [3]. Bioactive substances are chemical compounds obtained through microbiological or chemical synthesis that exhibit high physiological activity at low concentrations [4].

In recent years, biotechnologists have been closely focused on studying individual bioactive substances (BAS) in plant raw materials to develop functional preparations and dietary supplements containing antioxidants of natural or synthetic origin [5,6,7].

The human body is initially saturated with antioxidant substances in the early years of life, but over time, their contents decrease, and their natural effectiveness weakens [8,9]. There is a need to replenish antioxidant substances through food and additional sources, such as various antioxidant dietary supplements [10].

Antioxidants can protect cells through various mechanisms, such as:

1. Transforming reactive oxygen species (ROS) into non-radical forms (dependent on the involved antioxidant);
2. Interrupting the auto-oxidative chain reaction initiated by ROS;
3. Reducing localized oxygen concentrations [11].

Synthetic antioxidants include pharmaceuticals, functional food products, and dietary supplements that do not contain plant components. Synthetic antioxidants are used instead of natural ones because they provide higher stability and efficiency, lower cost, and widespread availability. The most frequently mentioned synthetic antioxidants in the food industry are butylated hydroxyanisole, butylated hydroxytoluene, propyl gallate, and tert-butylhydroquinone [12].

While synthetic antioxidants are widely used, safety concerns arise over time. Several published studies have suggested a link between prolonged intake of synthetic antioxidants and certain health issues [13]. High doses of synthetic antioxidants can cause DNA damage and premature aging [14]. Hence, the trend toward replacing synthetic antioxidants with natural ones is growing stronger.

Antioxidants derived from plant raw materials can react with reactive oxygen species, thereby protecting cellular structures from free-radical damage and contributing to the reduction of oxidative stress [15]. Substances such as vitamins, macro- and microelements, phenolic compounds, alkaloids, carotenoids, minerals, and enzymes found in fruits, vegetables, berries, and plants fall into the category of natural antioxidants.

Alkaloids are nitrogen-containing natural organic compounds that accumulate in all plant organs [16]. They have a positive impact on the cardiovascular, central nervous, and endocrine systems [17]. Notable alkaloids include caffeine, atropine, theobromine, and theophylline.

Carotenoids are plant pigments that impart bright yellow, orange, or red hues to plant materials. They possess antioxidant activity and immunomodulatory effects [18].

Phenolic acids represent aromatic secondary metabolites of plant origin. Phenolic acids have a protective function when consuming plant raw materials in the diet, especially in cardiovascular diseases and oncology. Examples of phenolic acid compounds include salicylic and gallic acids, as well as coumarins, catechins, quercetin, flavonoids, tannins, and others [19].

Considerable importance should be given to natural polyphenolic compounds, such as flavonoids, which exhibit pronounced biological properties. It has been established that flavonoids possess antibacterial and antiviral activity, anti-inflammatory, antiangiogenic, analgesic, and antiallergic effects, and hepatoprotective, cytostatic, apoptotic, estrogenic, and antiestrogenic properties [20]. They are characterized by capillary-strengthening, cardiotonic, spasmolytic, hypotensive, diuretic, choleretic, hepatoprotective, hemostatic, and anti-inflammatory actions [21].

Vitamins A, C, and E are important antioxidants that can reduce the body’s susceptibility to oxidative damage [22]. Some of these compounds are particularly crucial for human health, such as vitamins C and E, which act as antioxidants and perform essential functions in our bodies. They are not synthesized by the human body, so they must be obtained through food [23]. Vitamin E counters oxidative stress, preventing age-related diseases [24]. Vitamin C is a water-soluble antioxidant necessary for collagen synthesis, iron absorption, and epigenetic regulation. Vitamin C promotes proper immune system function, reduces inflammation, and prevents the occurrence and progression of various chronic and acute diseases [25].

Vitamin B_1_ is an essential component of nutrition, and a deficiency in this micronutrient underlies various diseases, especially nervous system disorders [26]. Vitamin B_1_ enhances plant resistance to environmental stress. Supplementary thiamine significantly increases the vitamin C content and the overall content of phenolic compounds in turnip varieties under drought conditions [27]. Vitamin B_6_ exhibits free-radical-scavenging activity and antioxidant effects [28]. Riboflavin (vitamin B_2_) is often referred to as the growth vitamin, controlling the protective antioxidant system in plants [29].

The common dandelion (*Taraxacum officinale*) is a perennial herbaceous plant of the Asteraceae family. It is a herbaceous plant that grows in North America, Europe, and Asia [30]. The dandelion was originally imported to America as a food crop and then spread to North America, New Zealand, Australia, South Africa, and India. It grows along roadsides, banks, lawns, pastures, and in areas with moist soils [31]. Owing to its wide range of nutrients, such as vitamins, minerals, polyphenols, flavonoids, and fatty acids, dandelion and its extracts exhibit anti-inflammatory and antioxidant effects. Additionally, they demonstrate anti-tumor and antimicrobial activity [32]. Dandelion alleviates inflammatory reactions in the intestine by restoring the balance of gut flora, suggesting that its anti-inflammatory effects are mediated by regulating gut microbial imbalance [33]. Traditionally, dandelion has been used to treat kidney, spleen, and liver diseases, as well as cardiovascular diseases, diabetes, and bacterial infections, and as an anti-inflammatory and diuretic agent [34].

Extracts from *T. officinale* are widely recognized as safe, and dandelion is considered a renewable resource, further enhancing its appeal as a natural food or medicinal product. Consequently, the popularity and usage of dandelion, its extracts, and key components are expected to continue growing. Recent articles report that flavonoids extracted from dandelion leaves and roots can be used as a functional supplement to starch to reduce the glycemic index [35]. Moreover, polysaccharides isolated from the entire dandelion plant during flowering exhibited prebiotic potential associated with microbiota composition modulation [36].

Greater Burdock (*Arctium lappa*) is a medicinal and edible homologous plant commonly known as burdock, belonging to the Asteraceae family [37]. Burdock is widespread in Europe [38], North America [39], China [40], Africa [41], and Asia [42], having both nutritional and medicinal value [43]. It is a globally cultivated medicinal and edible plant with predominantly phytochemical compounds and polysaccharides, possessing both nutritional and therapeutic properties [44]. The plant contains flavonoids and lignans, which are beneficial for treating high blood pressure, gout, thrombosis, hepatitis, and inflammatory diseases. Various biological properties, including antimutagenic, anticancer, and rejuvenating properties, are also attributed to its phenolic components [45]. Modern research reveals that *A. lappa* contains organic acids, flavonoids, terpenoids, lignans, and other components [46].

The roots, leaves, and fruits of burdock have varying therapeutic values and are widely used in some European and Asian countries. The fruits contain lignans and essential oils with anticancer and anti-inflammatory activity. The chemopreventive action of burdock fruits is linked to lignans such as arctiin and arctigenin [47]. *A. lappa* leaves exhibit high antioxidant activity and contain phenolic compounds, including phenolic acids, quercetin, quercitrin, and luteolin [48]. The main active ingredients extracted from burdock leaves are arctigenin, arctiin, coffee, and chlorogenic acids [49]. *A. lappa* roots contain a broad spectrum of bioactive substances such as polysaccharides, polyphenols, flavonoids, and volatile oils, contributing to their anti-inflammatory, antioxidant, antibacterial, antiviral, and other biological activities [50]. Phenolic acids (caffeic acid, chlorogenic acid, and cynarin), arctin, luteolin, and quercetin are found in burdock roots. The root possesses protective, anti-inflammatory, and free-radical-blocking activities that are attributed to chlorogenic acid derivatives. Burdock root contains inulin, essential oil, tannins, resins, carbohydrates, iron, calcium, and vitamin C, with its bitter taste resulting from linoleic and oleic acids [51].

A more in-depth and comprehensive study of local plants is required to extract bioactive substances used in the food industry. The aim of the research was to assess the content of water-soluble vitamins and flavonoids, radical-scavenging activity by ABTS, and antimicrobial activity of plant material from the Siberian Federal District, specifically *T. officinale* and *A. lappa*.

## 2. Results

### 2.1. Obtaining Extracts with High Contents of BAS

Extracts, depending on the solvent (extractant) used, can be alcoholic, aqueous, aqueous–alcoholic, essential, and CO_2_ extracts. Each method has its advantages and disadvantages. The optimal extractant for the extraction of BAS in order to obtain dietary supplements is an aqueous alcohol solvent. The parameters of extraction of plant raw materials were selected experimentally: temperature, solvent concentration, and incubation duration, as well as the hydromodule (ratio of raw materials to extractant). The results were evaluated using the antioxidant activity of the obtained extracts. The results are shown in Table 1.

By analyzing the results of the determination of antioxidant activity, it can be argued that the choice of an extractant should be carried out individually for each type of raw material. Within the framework of the conducted research, according to our own observations, a pattern was tracked during the experiment: the lighter the extract, the higher its antioxidant activity; the lower the alcohol concentration, the higher the concentrations of extracts of biologically active substances.

According to the results of this study, a high percentage of antioxidant activity was noted in all samples. The highest antioxidant activity in *Taraxacum officinale* was 85.51, and that for *Arctium lappa* was 88.97.

### 2.2. Study of Extracts for Antimicrobial Activity

Antimicrobial activity was studied for extracts based on plant raw materials with maximum antioxidant yield. The disc diffusion method was used. The results are presented in Table 2.

The water–alcohol extracts of the plant materials *Taraxacum officinale* and *Arctium lappa* demonstrated high antimicrobial activity against *Escherichia coli*. The disc diffusion method showed growth inhibition zones ranging from 10.5 to 13.5 mm for samples: 1, 4–6, and 28–30, which were extracts from dandelion roots. Inhibition zones of 10.5 to 15.5 mm were observed for samples: 37–42, 46–48, and 55–64, which were extracts from burdock roots.

The maximum diameter of the growth inhibition zone for dandelion was 13.5 mm (sample 28), while for burdock it was 15.5 mm (sample 58). Thus, the investigated plant materials *T. officinale* and *A. lappa* represent promising sources of bioactive substances (BAS) with antibacterial properties.

For the extraction of plant material from *A. lappa* and *T. officinale* with the highest possible content of BAS and a high antioxidant activity index, the optimal parameters are an incubation duration of 4 h, extraction temperature of 60 °C, and a plant material to solvent ratio of 1:10, respectively. The concentration of the extractant (ethyl alcohol) was 70% for *T. officinale* (sample 28), and the concentration of ethyl alcohol for *A. lappa* extraction was 40% (sample 58).

### 2.3. The Content of Water-Soluble Vitamins in Extracts with High Contents of Biologically Active Substances (BAS)

**Figure 1:**
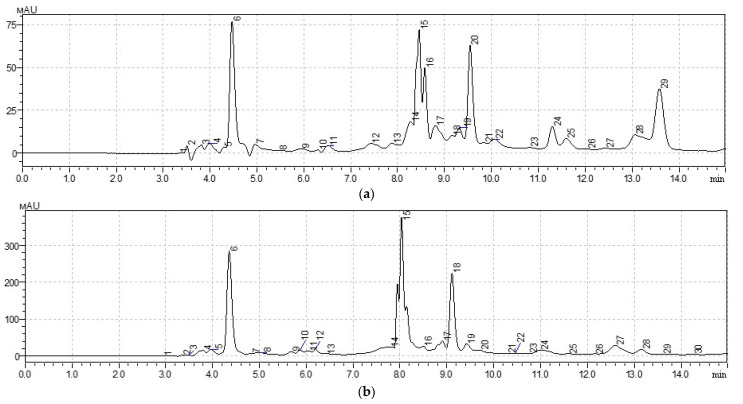
Chromatogram of HPLC analysis of plant extracts: (**a**) *Taraxacum officinale*: peak 6—vitamin C; peak 15, 16—vitamin B_1_; peak 20—vitamin B_3_; peak 24—vitamin B_6_; (**b**) *Arctium lappa*: peak 6—vitamin C; peak 15, 16—vitamin B*_1_*; peak 20—vitamin B_3_; peak 24—vitamin B_6_.

The results of the analysis of the determination of the qualitative and quantitative composition of water-soluble vitamins of groups B and C by high-efficiency liquid chromatography (HPLC) are shown in Figure 1, Table 3.

In the *Taraxacum officinale* sample, the study revealed a high content of pyridoxine (Vitamin B_6_) at 156.40 µg/mL. The presence of thiamine and niacin (vitamins B_1_ and B_3_), as well as vitamin C, was also noted. The *Arctium lappa* sample exhibited a significant concentration of B-vitamins, particularly thiamine (46.20 µg/mL) and pyridoxine (357.10 µg/mL). Additionally, niacin, pantothenic acid (Vitamin B_5_), and vitamin C were detected. It is noteworthy that sample 58 contains, on average, 13.02 times more B and C group vitamins than sample 28.

### 2.4. The Contents of Flavonoids in the Studied Extracts

**Figure 2:**
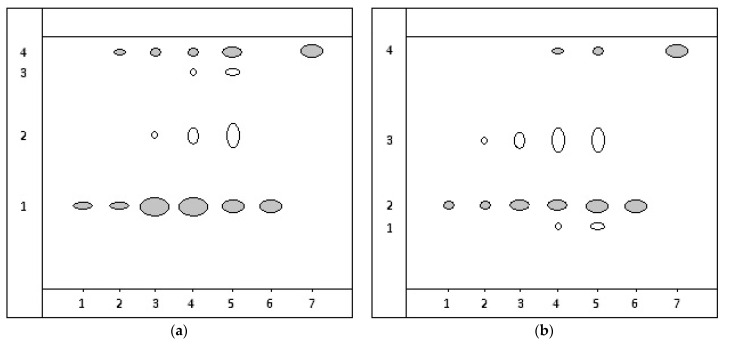
Chromatogram diagram of extraction from the root: (**a**) *Taraxacum officinale*, (**b**) *Arctium lappa*: on the start line of point 1—2 µL of extraction; point 2—3 µL; point 3—5 µL; 4—7 µL; point 5—10 µL; point 6—3 µL 0.1% standard rutin solution; point 7—3 µL 0.1% standard quercetin solution.

The results of the detection of flavonoids (rutin and quercetin) in an extract based on the plant raw materials *Taraxacum officinale* and *Arctium lappa* by TLC are presented in accordance with Figure 2, Table 4.

On the chromatogram of the extraction from the *Taraxacum officinale* root, when applying 2 µL, one spot was observed, which, based on the Rf value and the spot’s position, matched the rutin standard (Figure 2a, points 1, 6). When applying 3 µL of the extraction, two chromatographic zones appeared, identified as rutin and quercetin. Examining 5 µL of the extraction revealed 3 spots, with two being identified as rutin and quercetin. The unidentified compound is presumed to belong to flavonoids owing to its characteristic yellow–orange staining typical of flavones. Applying 7 and 10 µL of the extraction showed 4 chromatographic zones, with two being identified as rutin and quercetin (Table 4).

On the chromatogram of the extraction from the *Arctium lappa* root, applying 2 µL revealed one chromatographic zone identified as rutin, matching the standard solution (Figure 2b, points 1, 6). Applying 3 and 5 µL of the extraction showed 2 spots, with one being identified as rutin. The unidentified compound exhibited characteristic fluorescence under UV light after treating the plates with a 5% solution of aluminum chloride, indicating its flavonoid nature. Examining 7 and 10 µL of the extraction (Figure 2, points 4, 5) revealed 4 spots, with three being assigned to rutin, quercetin, and an unidentified flavonoid (Table 4).

## 3. Discussion

Plant material serves as a source of biologically active substances capable of preventing various diseases, including cardiovascular issues and cancer, and influencing the processes of skin aging and overall aging. This study focuses on two extracts based on the roots of *Taraxacum officinale* and *Arctium lappa*, examining their contents of BAS, specifically water-soluble vitamins and flavonoids. The investigated plant material also possesses antimicrobial activity and natural antioxidant compounds.

The conducted research demonstrates that extracts from dandelion and burdock roots exhibit low antimicrobial activity against *E. coli*, confirming the findings in the existing literature. In O. Kenny’s [52] study, a dandelion root extract showed antimicrobial activity against *Bacillus cereus* and *Staphylococcus aureus*, with no inhibition observed for *E. coli*. Research on water–alcohol extracts of burdock (*A. lappa*) and dandelion (*T. officinale*) by D. Ionescu and colleagues [53] identified antimicrobial activity against *Staphylococcus aureus, Escherichia coli,* and *Salmonella abony enterica*.

The results indicate a decrease in antioxidant activity with an increase in extract concentration, aligning with findings in N.P. Tiguntseva’s [54] study on extracting BAS from *Taraxacum officinale* using the water–alcohol extraction method. As alcohol concentration rises, the yield of biologically active compounds decreases to 9.6% for above-ground parts and 12.8% for roots. Minimal concentration leads to a 4% increase in the yield of extracted substances. The antioxidant activity under optimal extraction parameters was 85.51% for dandelion roots and 88.97% for burdock roots. In this study, a spectrophotometry method was used to measure the antioxidant capacity of plant extracts by capturing radicals using ABTS. Many scientists employ this method. Floegel A. and others [55] evaluated two of the most common radical-scavenging assays using the radicals 2,2′-azino-bis-3-ethylbenzthiazoline-6-sulphonic acid (ABTS) and 1,1-diphenyl-2-picrylhydrazyl (DPPH). The results showed that the ABTS assay better reflected the antioxidant content in various food products than the DPPH assay.

Our research reveals that extracts from *T. officinale* and *A. lappa* contain vitamins of the B and C groups. According to the obtained results, the dandelion root extract contains 10.19 mg/100 g of raw material of vitamin B_1_, 7.42 mg/100 g of vitamin B_3_, 156.40 mg/100 g of vitamin B_6_, and 0.46 mg/100 g of vitamin C. The burdock root extract contains 46.20 mg/100 g of raw material of vitamin B_1_, 23.70 mg/100 g of vitamin B_2_, 7.57 mg/100 g of vitamin B_5_, 357.10 mg/100 g of vitamin B_6_, and 21.87 mg/100 g of vitamin C. These findings align with the existing literature. For instance, in the study by W. Biel and colleagues [56], dandelion leaves were found to be rich in vitamin C (156.6 mg/100 g^–1^), thiamine (1.5 mg/100 g^–1^), riboflavin (3.0 mg/100 g^–1^), and niacin (11.8 mg/100 g^–1^). Another study by M. Martinez and others [57] determined that dried dandelion leaves, in addition to having high levels of carbohydrates, protein, fat, crude fiber, etc., also contain a significant amount of phenols, flavonoids, ascorbic acid (34.70 mg/100 g), β-carotene, chlorophyll, and antioxidants. In research conducted by Zakharov V.L. and team [58] on the content of biologically active substances (BAS) in the roots of medicinal herbaceous plants, traces of β-carotene, ascorbic acid (26.4 mg%), anthocyanins, carotenoids, flavanols (91.4 mg%), catechins, tannins, and coloring substances were found in *Taraxacum officinale*. The main active compounds identified in *A. lappa* include tannins, polyphenols, dietary fibers like inulin and lignans, and vitamins from groups B, C, K, and E, along with minerals (e.g., Na, Ca, Fe, Cu, Mg, P, K, and Zn). A study by X. Zhang and colleagues [59] confirms the presence of phenols, flavonoids (0.07–43.65 mg/g), carotenoids, vitamin C (0.18–13.02 mg/g), and in vitro antioxidant activity in burdock root.

In a study on the antioxidant activity of water-soluble vitamins, researcher Gliszczynska-Swigło A. [60] found that the vitamins thiamine (vitamin B_1_), folic acid (vitamin B_9_), pyridoxine, pyridoxal, and pyridoxamine (vitamin B_6_) are capable of scavenging the cation-radical ABTS+, although they reacted with it relatively slowly. The highest radical-scavenging activity was observed with thiamine, followed by forms of folic acid and vitamin B_6_.

According to the literature, flavonoids are also known to contribute to health improvement, partly owing to their antioxidant properties, as demonstrated in many in vitro studies [61]. In extracts from *T. officinale* and *A. lappa*, the presence of the medically significant anti-diabetic flavonoid quercetin was identified. In the study by T.M.A. Moro and colleagues [62], cynarin, chlorogenic acid, caffeic acid, and quercetin were identified as the main metabolites present in extracts from *A. lappa* roots. When investigating the hydroethanolic extract of *Arctium lappa* root, Predes F.S. and co-authors [63] identified the presence of compounds such as arctigenin, quercetin, chlorogenic acid, and caffeic acid. In a study by K. Schütz and others [64], phenolic acids and flavonoids were extracted from dandelion roots and leaves (*Taraxacum officinale* WEB. ex WIGG.). The extracts contained mainly chicoric acid, and glycosides of quercetin were also detected.

Rutin belongs to the flavonoid family. It is a molecule of quercetin with the addition of a disaccharide (rutinose and glucose). Thanks to its anti-inflammatory and antioxidant effects, it has gained significant importance in the pharmaceutical industry, and many drugs registered worldwide contain rutin [65,66]. Rutin is quite commonly found in plant material, and notably, ethanol extracts are used for its commercial purification [67]. In the work by de Souza A.R.C. and others [68], high contents of chlorogenic acid (1.84%) and rutin (1.46%) were found in the ethanol extract of *Arctium lappa* leaves, as well as significant concentrations of phytol, lupeol, and amyrin. When studying the antioxidant potential of Taraxacum officinale tincture, Epure A. and others [69] identified chicoric acid in the highest quantity in the polyphenolic composition, but other phenolic acids (protocatechuic, vanillic, syringic, and ferulic acids) were also present, along with flavonoids (rutin, quercitrin, luteolin, and apigenin).

Based on the results of our study, it can be inferred that quercetin and vitamin C play active roles in demonstrating antioxidant activity. Researchers Rusmana D. and others [70] conducted an antioxidant analysis of Phyllanthus niruri extract, as well as rutin and quercetin compounds, which are flavonoids with therapeutic properties. They found that the P. niruri extract and quercetin exhibited significant activity in reducing ABTS, with quercetin showing higher antioxidant activity than the extract. It was also found that rutin was not effective in reducing the ABTS radical. In the work by Duenas M. and co-authors [71], high antioxidant activity of metabolites was detected in the ABTS analysis, suggesting that quercetin derivatives may act as potential radical scavengers under physiological conditions. In this study, a solution of vitamin C was used as a control for determining the antioxidant activity of plant extracts, as it has demonstrated antioxidant activity [72,73]. In the study by Kim D.-O. and others [74], the antioxidant potential of phenolic compounds was assessed using vitamin C as a control. The authors also found that in the ABTS analysis, the antioxidant capacity of phenolic compounds was ranked as follows: gallic acid > quercetin > epicatechin > catechin > vitamin C > rutin > chlorogenic acid > trolox.

## 4. Materials and Methods

**Figure 3:**
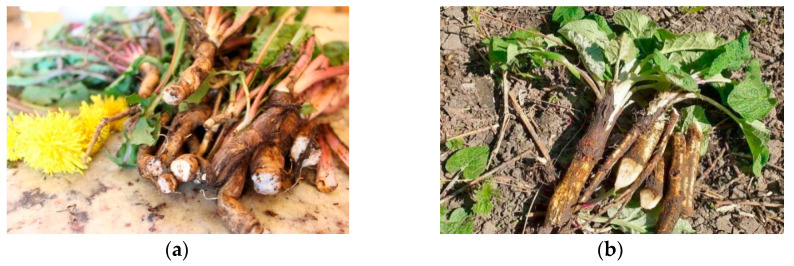
The appearance of the objects of study: (**a**) *T. officinale*; (**b**) *A. lappa*.

The objects of research were plant raw materials of the root part of *Taraxacum officinale Arctium lappa*, collected in the Kemerovo region. The general appearance of the objects is shown in Figure 3.

Extraction of plant components. The roots of *Taraxacum officinale* and *Arctium lappa* were crushed to a size with a square side equal to 5 mm and then ground in a porcelain mortar with a pestle. An aqueous alcohol solvent was determined to be the optimal extractant for the extraction of bioactive substances (BAS). Ethanol concentrations of 40%, 70%, and 96% were utilized for extraction. According to the literature, optimal recovery conditions for tannins suggest the use of 40% ethanol as an extractant [75,76], while maximum extraction of flavonoids is achieved with 70% ethanol [77,78]. Additionally, 96% ethanol is employed for extracting major secondary metabolites such as alkaloids, tannins, flavonoids, saponins, steroids, and phlobatannins [79,80]. Hydromodule (ratio of vegetable raw materials–solvent)—1:10; 1:20; 1:30. Extraction temperature: 30 and 60 °C. The extraction time varied from 0.5 h to 4 h. Then, the resulting mixture was passed through desalted filters [81]. The extraction parameters are presented in Table 5.

The determination of antioxidant activity was conducted using a spectrophotometer with an original solution of 2,2′-azino-bis-[3-ethylbenzthiazoline sulfonate] (ABTS). Initial solutions of ABTS (7 mmol × L^–1^) and potassium persulfate (140 mmol × L^–1^) were prepared. The reaction mixture was prepared by combining these two initial solutions in equal volumes and leaving them for 16 h at room temperature in the dark. The final solution was diluted by mixing 5 mL of ABTS with 88 μm methanol to achieve an optical density of 0.70 ± 0.10 at 734 nm. In test tubes, 3 mL of ABTS solution and 1 mL of plant extract were added. After 8 min of incubation at 21 °C, the mixture was transferred to a quartz cuvette, and the optical density was measured at a wavelength of 734 nm [82]. Distilled water was used as a negative control. Vitamin C solution was performed as a positive control. For this purpose, a solution with a substance concentration of 1000 mmol/L was prepared.

The radical-scavenging activity for the samples was expressed as the percentage of ABTS^+^ radical scavenging according to Formula (1):

(1) $$X=\frac{\left(A_{0}-A_{1}\right)}{A_{0}}\cdot 100\%,$$

where: X—percentage of ABTS^+^ radical capture, %;

A_0_—optical density of the control tube;

A_1_—optical density of the samples.

For the investigation of antimicrobial activity in plant-based extracts, disc diffusion following the CLSI guidelines [83] was employed. To initiate the assay, the model microorganism *Escherichia coli* (B-8208, All-Russian State Collection of Microorganism Strains) was cultured overnight from a single colony in meat-peptone agar (MPA; Lenreaktiv, Russia) at 37 °C and 200 rpm. A suspension of the E. coli was then prepared in sterile 0.9% NaCl solution (Lenreaktiv, Russia) to an optical density of 0.5 on the McFarland scale (1.5 × 10^8^ Colony Forming Units CFU/mL), utilizing a densitometer (Densichek plus, Sendle, Russia). The culture was subsequently spread-plated on Muller Hinton agar (Himedia, Mumbai, India). Processed extract disks with a diameter of 6 mm were placed on the agar surface. Each dish contained 5 disks with a single extract to minimize variations in the size of the inoculum, environment, and incubation conditions [84]. The dishes were then incubated at 37 °C for 24 h, and the results were interpreted by measuring the diameter of the inhibition zone with millimeter precision. Distilled water served as the negative control, while a tetracycline antibiotic solution (Himedia, Mumbai, India) served as the positive control, with an antibiotic concentration of 10 µg.

Determination of Vitamin Content: Qualitative and quantitative analysis of water-soluble (B and C groups) vitamins was performed using high-performance liquid chromatography (HPLC) [85]. The study was conducted on an LC-20 Prominence chromatograph with a diode array detector Shimadzu SPD20MA, a fluorescence detector RF-20Axs, and an integrated post-column derivatization system Phenomenex Gemini C-18 250 × 4.6 mm (Kyoto, Japan, Shimadzu).

Conditions for the qualitative and quantitative composition of water-soluble vitamins:

- Column thermostat temperature: 25 °C.
- Flow rate of the mobile phase: 800 mm/min.
- Sample injection volume: 20 mm.
- Gradient elution mode was used for separation (mobile phase A—a solution of potassium dihydrogen phosphate at a molar concentration of 0.05 mol/dm^3^ and pH 3, mobile phase B—acetonitrile).

The determination of flavonoid contents in extracts based on the plant raw materials *Taraxacum officinale* and *Arctium lappa* was determined using the standard method of thin-layer chromatography (TLC) [86]. The analysis was carried out using TLC plates and a solution of n-butanol with glacial acetic acid and the addition of distilled water (in a ratio of 4:1:5). Volumes of 2, 3, 5, 7, 10 µL of the studied extract and 3 µL of 0.1% solutions of standard samples of rutin and quercitin were simultaneously applied to the plastic. To obtain standard solutions of rutin and quercetin, stock solutions of dimethyl sulfoxide (Lenreaktiv, St Petersburg, Russia) were prepared and diluted with distilled water [15]. After TLC analysis, the spots were exposed under a lamp with ultraviolet light at 254 nm. The value of the relative velocity of movement of flavonoids (R*f*) was determined using Formula (2):

(2) $$R_{f}=\frac{x}{L}$$

where: x—the distance covered by the substance, cm;

L—the distance covered by the eluent.

Statistical analysis was conducted using Student’s *t*-test for paired values to determine significant deviations from control values. Differences were considered statistically significant at *p* < 0.05. The tables and figures show the arithmetic mean values of the indicators under study. All the experiments were carried out in triplicate.

The equipment for the study was provided by the Instrumental Methods of Analysis in Applied Biotechnology Center at Kemerovo State University.

The work was carried out within the framework of the RNF project “Fundamentals of obtaining bioactive substances from medicinal plants of the Siberian region and creating phytogenic feed additives of immunomodulatory action on their basis”, No. 23-16-00113, dated 15 May 2023.

## Figures and Tables

**Table 1:** Results of antioxidant activity.

Sample	Antioxidant Activity (Mean ± SD), %	Sample	Antioxidant Activity (Mean ± SD), %
Water	0	Vitamin C (1000 mmol/L)	50.13 ± 1.03
1	69.62 ± 1.43 * Tst = 11.06 *p* = 0.001	37	87.21 ± 1.82 * Tst = 17.73 *p* = 0.0004
2	64.06 ± 1.32 * Tst = 8.32 *p* = 0.004	38	85.13 ± 1.75 * Tst = 17.24 *p* = 0.0004
3	61.15 ± 1.26 * Tst = 6.77 *p* = 0.01	39	83.72 ± 1.72 * Tst = 16.75 *p* = 0.0005
4	83.30 ± 1.76 * Tst = 16.27 *p* = 0.001	40	81.11 ± 1.67 * Tst = 15.79 *p* = 0.001
5	81.15 ± 1.67 * Tst = 15.81 *p* = 0.001	41	80.56 ± 1.66 * Tst = 15.58 *p* = 0.001
6	78.97 ± 1.63 * Tst = 14.96 *p* = 0.001	42	76.20 ± 1.57 * Tst = 13.88 *p* = 0.001
7	63.59 ± 1.31 * Tst = 8.08 *p* = 0.004	43	53.59 ± 1.10 Tst = 1.63 *p* = 0.20
8	62.39 ± 1.29 * Tst = 7.43 *p* = 0.01	44	49.66 ± 1.02
9	59.44 ± 1.22 * Tst = 5.83 *p* = 0.01	45	47.91 ± 0.99
10	66.07 ± 1.36 * Tst = 9.34 *p* = 0.003	46	77.26 ± 1.59 * Tst = 14.32 *p* = 0.001
11	63.42 ± 1.31 * Tst = 7.98 *p* = 0.004	47	76.32 ± 1.57 * Tst = 13.95 *p* = 0.001
12	62.18 ± 1.28 * Tst = 7.33 *p* = 0.01	48	74.57 ± 1.54 * Tst = 13.19 *p* = 0.001
13	49.36 ± 1.02	49	35.17 ± 0.72
14	47.91 ± 0.99	50	32.44 ± 0.67
15	47.05 ± 0.97	51	31.37 ± 0.65
16	48.55 ± 1.00	52	39.53 ± 0.81
17	46.67 ± 0.96	53	36.84 ± 0.76
18	46.03 ± 0.95	54	32.78 ± 0.68
19	63.42 ± 1.31 * Tst = 63.42 *p* = 0.004	55	72.22 ± 1.49 * Tst = 12.20 *p* = 0.001
20	60.90 ± 1.25 * Tst = 6.65 *p* = 0.01	56	70.90 ± 1.46 * Tst = 11.62 *p* = 0.001
21	56.84 ± 1.17 * Tst = 4.30 *p* = 0.02	57	68.76 ± 1.42 * Tst = 10.62 *p* = 0.002
22	67.05 ± 1.38 * Tst = 9.83 *p* = 0.002	58	88.97 ± 1.83 * Tst = 18.50 *p* = 0.0003
23	51.11 ± 1.05 Tst = 0.47 *p* = 0.67	59	87.56 ± 1.80 * Tst = 18.05 *p* = 0.0004
24	48.29 ± 0.99	60	85.85 ± 1.77 * Tst = 17.44 *p* = 0.0004
25	58.33 ± 1.20 * Tst = 5.19 *p* = 0.01	61	82.56 ± 1.70 * Tst = 17.82 *p* = 0.0004
26	49.15 ± 1.01	62	79.10 ± 1.63 * Tst = 15.02 *p* = 0.001
27	44.74 ± 0.92	63	74.96 ± 1.54 * Tst = 13.40 *p* = 0.001
28	85.51 ± 1.76 * Tst = 17.35 *p* = 0.0004	64	67.74 ± 1.40 * Tst = 10.13 *p* = 0.002
29	82.22 ± 1.69 * Tst = 16.21 *p* = 0.001	65	65.94 ± 1.36 * Tst = 9.27 *p* = 0.003
30	80.38 ± 1.66 * Tst = 15.48 *p* = 0.001	66	62.35 ± 1.28 * Tst = 7.44 *p* = 0.01
31	62.05 ± 1.28 * Tst = 7.26 *p* = 0.01	67	32.69 ± 0.67
32	59.87 ± 1.23 * Tst = 6.07 *p* = 0.01	68	28.93 ± 0.60
33	55.77 ± 1.15 * Tst = 3.65 *p* = 0.04	69	28.42 ± 0.59
34	63.42 ± 1.31 * Tst = 7.98 *p* = 0.004	70	54.49 ± 1.12 Tst = 2.04 *p* = 0.13
35	50.68 ± 1.04 Tst = 0.26 *p* = 0.81	71	47.22 ± 0.97
36	48.33 ± 1.00	72	44.19 ± 0.91

SD—standard deviation; *—Statistically significant results. The observed differences are statistically significant (significance level *p* < 0.05); Tst—Student’s *t*-test value when comparing the studied treatment option with the control vitamin C (1000 mmol/L).

**Table 2:** The results of the analysis of the antimicrobial activity of plant extracts against the test pathogen *E. coli*. Disc size = 6 mm; S = low susceptibility (7.0–10.0 mm); S+ = susceptibility (10.5–15.0 mm); S++ = high susceptibility (15.5–18.0 mm); R = resistant (0 mm).

Sample	Inhibition Zone (Mean ± SD), mm	Response	Sample	Inhibition Zone (Mean ± SD), mm	Response
Water	0	R	Tetracycline (10 µg)	13.0 ± 0.3	S+
1	11.0 ± 0.2	S+	37	12.0 ± 0.3	S+
2	10.0 ± 0.2	S	38	12.0 ± 0.3	S+
3	10.0 ± 0.2	S	39	11.5 ± 0.2	S+
4	13.0 ± 0.3	S+	40	13.5 ± 0.3 Tst = 1.18 *p* = 0.32	S+
5	11.0 ± 0.2	S+	41	13.0 ± 0.3	S+
6	11.0 ± 0.2	S+	42	11.5 ± 0.2	S+
7	10.0 ± 0.2	S	43	10.0 ± 0.2	S
8	10.0 ± 0.2	S	44	9.5 ± 0.2	S
9	10.0 ± 0.2	S	45	8.5 ± 0.2	S
10	9.5 ± 0.2	S	46	12.0 ± 0.3	S+
11	9.0 ± 0.2	S	47	12.0 ± 0.3	S+
12	9.0 ± 0.2	S	48	11.5 ± 0.2	S+
13	9.0 ± 0.2	S	49	9.0 ± 0.2	S
14	8.5 ± 0.2	S	50	6.5 ± 0.1	S
15	9.0 ± 0.2	S	51	0	R
16	8.0 ± 0.2	S	52	7.5 ± 0.2	S
17	7.0 ± 0.1	S	53	7.5 ± 0.2	S
18	0	R	54	6.5 ± 0.1	S
19	10.0 ± 0.2	S	55	11.5 ± 0.2	S+
20	10.0 ± 0.2	S	56	12.5 ± 0.3	S+
21	9.0 ± 0.2	S	57	12.5 ± 0.3	S+
22	9.5 ± 0.2	S	58	15.5 ± 0.4 * Tst = 5.89 *p* = 0.01	S++
23	9.0 ± 0.2	S	59	13.0 ± 0.3	S+
24	8.5 ± 0.2	S	60	11.5 ± 0.2	S+
25	9.0 ± 0.2	S	61	11.5 ± 0.2	S+
26	8.5 ± 0.2	S	62	10.5 ± 0.2	S+
27	0	R	63	11.5 ± 0.2	S+
28	13.5 ± 0.3 Tst = 1.18 *p* = 0.32	S+	64	10.5 ± 0.2	S+
29	11.5 ± 0.2	S+	65	9.5 ± 0.2	S
30	10.5 ± 0.2	S+	66	9.5 ± 0.2	S
31	10.0 ± 0.2	S	67	6.5 ± 0.1	S
32	9.5 ± 0.2	S	68	0	R
33	9.0 ± 0.2	S	69	0	R
34	10.0 ± 0.2	S	70	9.5 ± 0.2	S
35	9.0 ± 0.2	S	71	9.0 ± 0.2	S
36	8.0 ± 0.2	S	72	7.5 ± 0.2	S

SD—standard deviation; *—Statistically significant results compared with positive control (tetracycline). The observed differences are statistically significant (significance level *p* < 0.05); Tst—Student’s *t*-test value when comparing the studied treatment option with the control tetracycline (10 µg).

**Table 3:** The quantitative contents of water-soluble vitamins according to HPLC analysis.

Name of the Vitamin	Amount of Substance, ug/mL of Extract	
	Sample 28 (*Taraxacum officinale*)	Sample 58 (*Arctium lappa*)
Vitamin B_1_	10.19	46.20
Vitamin B_2_	–	–
Vitamin B_3_	7.42	23.70
Vitamin B_5_	–	7.57
Vitamin B_6_	156.40	357.10
Vitamin C	0.46	21.87

**Table 4:** Parameters of chromatographic zone separation on the chromatogram of extraction from the root of *Taraxacum officinale* and *Arctium lappa*.

*Taraxacum officinale*			*Arctium lappa*		
Spot No.	R*f* ± 0.02	Corresponding Compound	Spot No.	R*f* ± 0.02	Corresponding Compound
1	0.54	Rutin	1	0.34	n/i
2	0.73	n/i	2	0.51	Rutin
3	0.91	n/i	3	0.71	n/i
4	0.96	Quercetin	4	0.99	Quercetin

n/i—not identified.

**Table 5:** Extraction parameters of plant extracts.

Sample №	Incubation Duration, h	Alcohol Concentration, %	Temperature, °C	Hydromodule, Raw Material:Alcohol
*Taraxacum officinale* root				
1	0.5	40	30	1:10
2	0.5	40	30	1:20
3	0.5	40	30	1:30
4	0.5	40	60	1:10
5	0.5	40	60	1:20
6	0.5	40	60	1:30
7	0.5	70	30	1:10
8	0.5	70	30	1:20
9	0.5	70	30	1:30
10	0.5	70	60	1:10
11	0.5	70	60	1:20
12	0.5	70	60	1:30
13	0.5	96	30	1:10
14	0.5	96	30	1:20
15	0.5	96	30	1:30
16	0.5	96	60	1:10
17	0.5	96	60	1:20
18	0.5	96	60	1:30
19	4.0	40	30	1:10
20	4.0	40	30	1:20
21	4.0	40	30	1:30
22	4.0	40	60	1:10
23	4.0	40	60	1:20
24	4.0	40	60	1:30
25	4.0	70	30	1:10
26	4.0	70	30	1:20
27	4.0	70	30	1:30
28	4.0	70	60	1:10
29	4.0	70	60	1:20
30	4.0	70	60	1:30
31	4.0	96	30	1:10
32	4.0	96	30	1:20
33	4.0	96	30	1:30
34	4.0	96	60	1:10
35	4.0	96	60	1:20
36	4.0	96	60	1:30
*Arctium lappa* root				
37	0.5	40	30	1:10
38	0.5	40	30	1:20
39	0.5	40	30	1:30
40	0.5	40	60	1:10
41	0.5	40	60	1:20
42	0.5	40	60	1:30
43	0.5	70	30	1:10
44	0.5	70	30	1:20
45	0.5	70	30	1:30
46	0.5	70	60	1:10
47	0.5	70	60	1:20
48	0.5	70	60	1:30
49	0.5	96	30	1:10
50	0.5	96	30	1:20
51	0.5	96	30	1:30
52	0.5	96	60	1:10
53	0.5	96	60	1:20
54	0.5	96	60	1:30
55	4.0	40	30	1:10
56	4.0	40	30	1:20
57	4.0	40	30	1:30
58	4.0	40	60	1:10
59	4.0	40	60	1:20
60	4.0	40	60	1:30
61	4.0	70	30	1:10
62	4.0	70	30	1:20
63	4.0	70	30	1:30
64	4.0	70	60	1:10
65	4.0	70	60	1:20
66	4.0	70	60	1:30
67	4.0	96	30	1:10
68	4.0	96	30	1:20
69	4.0	96	30	1:30
70	4.0	96	60	1:10
71	4.0	96	60	1:20
72	4.0	96	60	1:30

## Data Availability

Data are contained within the article.
